# Hemostatic Collagen Sponge with High Porosity Promotes the Proliferation and Adhesion of Fibroblasts and Osteoblasts

**DOI:** 10.3390/ijms24097749

**Published:** 2023-04-24

**Authors:** Emira D’Amico, Tania Vanessa Pierfelice, Stefania Lepore, Giovanna Iezzi, Camillo D’Arcangelo, Adriano Piattelli, Ugo Covani, Morena Petrini

**Affiliations:** 1 Department of Medical, Oral and Biotechnological Sciences, University G. d’Annunzio of Chieti-Pescara, 66100 Chieti, Italy; 2 School of Dentistry, Saint Camillus International University of Health and Medical Sciences, Via di, Sant’Alessandro 8, 00131 Rome, Italy; 3 Facultad de Medicina, UCAM Universidad Catolica San Antonio de Murcia, 30107 Murcia, Spain; 4 Istituto Stomatologico Toscano, Via Aurelia 335, 55041 Lido di Camaiore, Italy

**Keywords:** collagen sponge, human gingival fibroblasts, human oral osteoblasts, osteoinductive, osteocalcin, phosphatase alkaline, porosity, regeneration

## Abstract

The use of biomaterial for tissue repair involves the interaction between materials and cells, and the coagulum formation represents the first step of tissue healing. This process is particularly critical in the oral cavity, where the wounds are immediately subjected to the masticatory mechanical stress, saliva invasion, and bacterial attack. Therefore, the present study aimed to explore the structural features and the biological activities of a hemostatic collagen sponge on human gingival fibroblasts (HGFs) and human oral osteoblasts (HOBs). The microstructure of the collagen sponge was characterized by a scanning electron microscope (SEM) and histological analysis. The porosity was also calculated. To investigate biological activities, HGFs and HOBs were cultured on the collagen sponges, and their adhesion was observed at SEM on the third day, while cell viability was investigated at the third and seventh days by Tetrazolium (MTT) assay. For osteoblasts seeded on collagen sponge the mineralization ability was also evaluated by alkaline phosphatase (ALP) assay at the seventh day, and by Alizarin red staining on the 14th. Furthermore, the gene expression of ALP and osteocalcin (OCN) was investigated after 3, 7 and 14 days. SEM images of the sponge without cells showed a highly porous 3D structure, confirmed by the measurement of porosity that was more than 90%. The samples cultured were characterized by cells uniformly distributed and adhered to the sponge surface. Proliferation ended up being promoted, as well as the mineralization ability of the osteoblasts, mainly at the mature stage. In conclusion, this collagen sponge could have a potential use for tissue healing.

## 1. Introduction

Surgical incisions are among the conditions in which wound healing plays a critical role in restoring the integrity of skin [1]. However, in the case of infections, scarring, genetic disorders, and various pathologies, this process could be affected or arrested, leading to chronic wounds [2] The re-epithelization represents the last healing phase, and without its occurrence, the skin cannot be healed. Thus, any impairments in the phases that come before may contribute to the failure of the re-epithelization [3]. Considering that hemostasis represents the first stage of the wound healing process, to limit blood loss from skin lesions, various hemostatic agents have been introduced [4,5]. Heterogeneous collagen is the insoluble fibrous protein that composes the extracellular matrix (ECM), thus it exhibits excellent biocompatibility. Moreover, collagen fibers can acquire great tensile strength and stability by cross-linking and self-aggregation. Together, these properties make exogenous collagen ideal for biomaterial applications [6,7]. Among collagen-based materials, sponges have been widely used as hemostatic tools thanks to their feasibility in inducing the regeneration of the skin, connective tissue, trachea, esophagus, adipose tissue, and peripheral nerves [8,9]. Collagen is also employed in the form of membranes in guided tissue regeneration (GTR) procedures to stabilize the coagulum, to prevent the invasion of the surgical site by cells with higher turnover, and to avoid the arriving of exogenous particles and microorganisms at the site [10]. In specific situations, deep injuries involve skin repair, and soft and hard tissue remodeling. Therefore, it is fundamental to favor all three processes by providing a substrate able to act as a hemostatic agent, in order to promote the proliferation of viable fibroblasts and provide a template for new tissue growth. In the case of surgical sites in the oral cavity, a first intention closure of the wound is mandatory. However, it is not always possible, and in that case, the use of hemostatic tools that could protect the coagulum from masticatory mechanical stress and bacterial infection is fundamental [11,12]. Consequently, the ideal hemostatic tool permits not only immediate bleeding control and coagulum formation, but also interaction with the host cells to permit rapid wound closure. In particular, repairing bone defects requires a material with a structure that makes it a favorable microenvironment for the repopulation of osteoblasts, but it could also promote the adhesion of other cells, such as fibroblasts that secrete signals such as fibroblast growth factor 2 (FGF2) that affect osteoblasts proliferation [13,14]. Therefore, the present study aimed to explore both the structural features and the biological activities of a collagen sponge that was until now used as a hemostatic tool. Firstly, a characterization of the collagen sponge microstructure was performed, and considering the fundamental role of the pore size in the scaffold for cellular interaction [15], the porosity was also calculated. The biological properties of collagen sponges were first of all investigated on a population of cells mainly involved in the oral wound healing process, such as gingival fibroblasts, with regard to the vitality and the adhesion at 3 and 7 days post-seeding. The collagen sponge was then investigated for biological properties on oral osteoblasts. The vitality and the adhesion were studied at 3 and 7 days after seeding, the activity of the ALP enzyme was investigated at 7 days, the mineralization activity was investigated at 14 days, and gene expression of the mineralization-related genes were investigated at 3, 7, and 14 days.

## 2. Results

### 2.1. Characterization of Collagen Sponge

#### 2.1.1. Microstructure and Porosity

**Figure 1:**
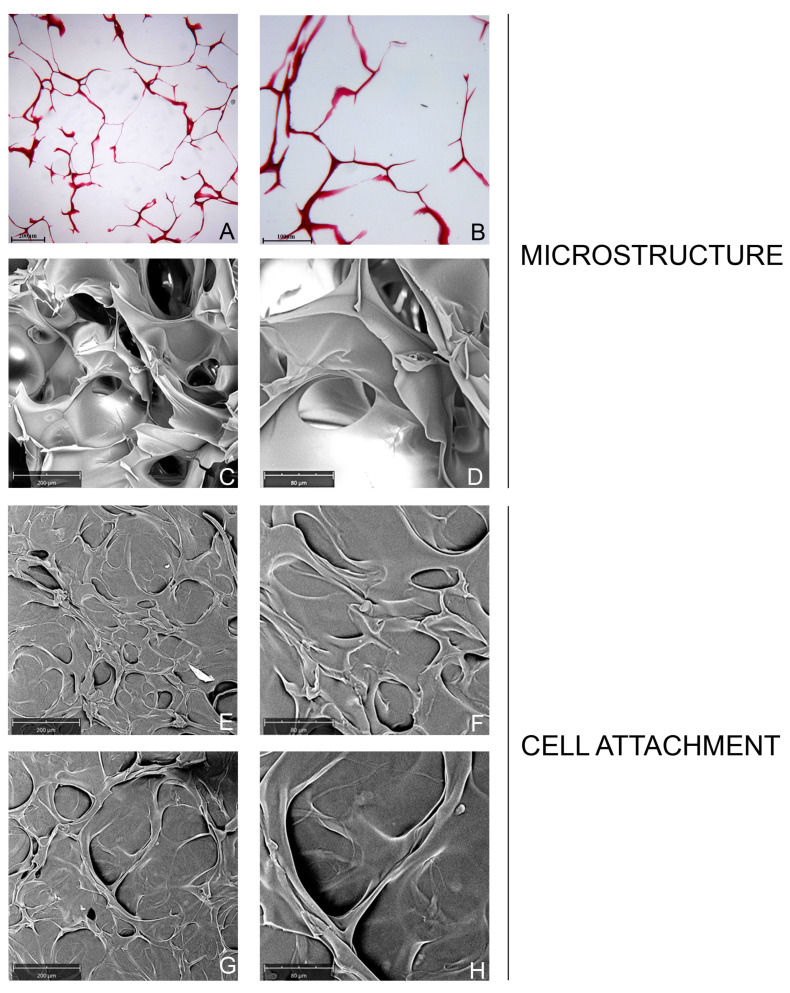
Characterization of the microstructure of the collagen sponge and cell attachment. (**A**,**B**): Histological analysis of the structure of collagen sponge scaffolds. Mag = 40×, 100×. (**C**,**D**): Scanning electron micrograph (SEM) images of the 3D structure of collagen sponge scaffolds. Mag = 390×, 1000×. (**E**–**H**): SEM) images of seeded collagen sponge scaffolds. The SEM micrographs of the gingival fibroblasts (**E**,**F**) (and oral osteoblasts (**G**,**H**)) adhering to the collagen-sponge after 3 days of seeding. (Mag = 390×, Mag = 1000×).

The histological observations showed the high porosity of the collagen sponge with a honeycomb-like structure (Figure 1A,B). The SEM image showed the 3D structure of the collagen-sponge with a honeycomb architecture (Figure 1C,D). The collagen scaffold exhibited a quite regular and homogenous highly porous structure with a porosity of 97.52%, and the pore size was on average 244.69 ± 80.01 µm (Table 1). Furthermore, the histological measurements of the porous size confirmed the range observed by SEM.

#### 2.1.2. Cell Attachment and Cell Morphology

In this study, the adhesion of cells on a collagen-sponge was observed at SEM (Figure 1E–H). At 3 days post seeding, both fibroblasts (Figure 1E,F) and osteoblasts (Figure 1G,H) appeared tightly connected, and no dead cells were present, indicating an excellent cell viability of the scaffold. The SEM observation showed that the cells were uniformly distributed on the collagen sponge. The cells were also distributed on the pore walls. Cells were grown on collagen surfaces and formed a dense multilayer-sheet of flattened cells. The morphology of the HGFs and HOBs was also evaluated at SEM (Figure 1E–H). Both types of adhered cells on the collagen-sponge exhibited a good morphology. The shape appeared as a spindle, and the attached cells created a dense network on the porous structure surface. At 7 days post-seeding, obtaining the images at SEM was not been possible because of the natural degradation of the collagen sponge that occurred. 

### 2.2. Biological Activity

#### 2.2.1. Cell Viability

**Figure 2:**
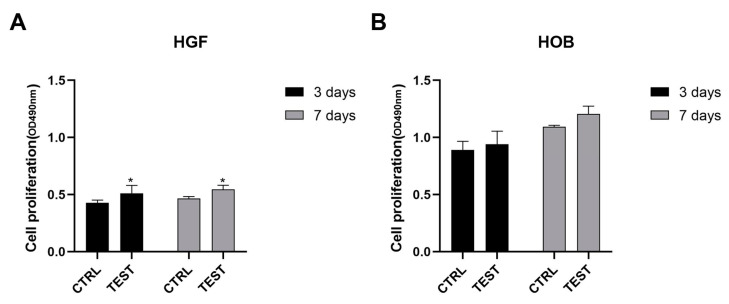
Cell viability of collagen-sponge after 3 and 7 days of hGFs (**A**) and hOBs (**B**) cultures. (* *p* < 0.05 compared to control).

The biocompatibility properties of the collagen-scaffold was assessed by a cell viability assay, as shown in Figure 2. After culturing for 3 and 7 days, gingival fibroblasts (Figure 2A) seeded on sponge-like collagen proliferated in a statistically significant way (*p* < 0.05) with respect to the control. Although the collagen sponge enhanced oral osteoblast proliferation at 3 and 7 days of culture (Figure 2B), the result was insignificant. At 3 days, the gingival fibroblasts showed the highest proliferation rate, while oral osteoblasts had the highest at 7 days. Furthermore, there was no significant difference in the growth of cells between 3 and 7 days. 

#### 2.2.2. Mineralization Activity of Osteoblasts 

**Figure 3:**
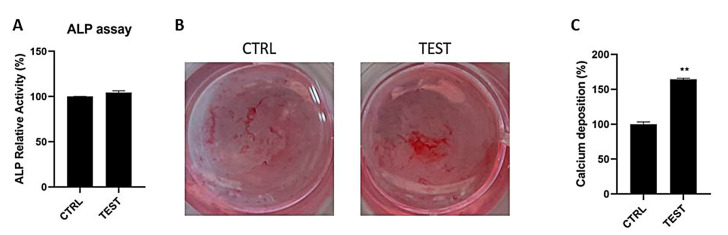
ALP activity of osteoblasts cultured for 7 days (**A**) Alizarin red staining on osteoblasts cultured for 14 days (**B**), calcium deposition quantification at 14 days (**C**). (** *p* < 0.01 compared to control).

Osteoblasts in the tested and control groups showed a similar pattern of levels of ALP activity (Figure 3A). At 7 days of culture, the enzymatic activity became enhanced when cells were seeded on the collagen group, but no significant differences in activity between the study groups were found. After 14 days of culture, the calcium mineralization of osteoblasts was demonstrated by Alizarin red staining and quantified by adding CPC. Figure 3B showed more mineralized nodules in osteoblasts seeded on a collagen sponge (test group) compared to the control. It is possible to detect the difference by observing the brighter red color in the collagen sponge. This qualitative data was confirmed by the quantification of calcium deposition that ended up significantly higher (*p* < 0.01) in the collagen group (Figure 3C). 

#### 2.2.3. Gene Expression of ALP and OCN in Osteoblasts

**Figure 4:**
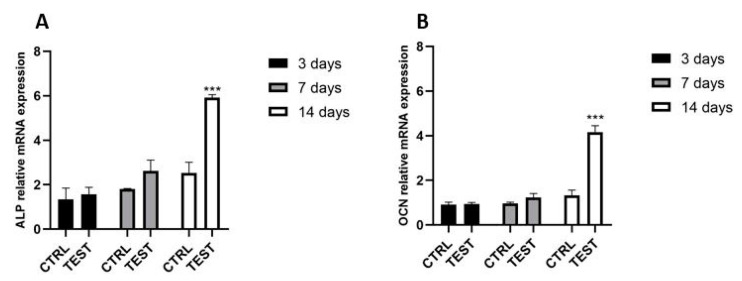
Real-time PCR of osteoblasts seeded on the control surface and on the collagen scaffold for genes encoding alkaline phosphatase (**A**), and osteocalcin (**B**) at 3, 7 and 14 days post seeding. (*** *p* < 0.001 compared to control).

The effect of the collagen sponge on osteoblasts was investigated at 3, 7 and 14-days post-seeding utilizing real-time PCR (Figure 4). It was first observed that neither the control nor the test group had any influence on genes encoding phosphatase alkaline (ALP), and osteocalcin (OCN) at the early time point of 3 days post-seeding (Figure 4A). However, after 7 days of culture, ALP slightly increased compared to control. At 14 days post-seeding, it was found that the increment of mRNA levels for ALP resulted in significantly higher levels than the control. Similarly, for osteoblasts seeded on a collagen sponge, the OCN mRNA levels resulted upregulated up to two-fold at 14 days post seeding, while at 3 and 7 days there were any significative enhancements compared to the control (Figure 4B).

## 3. Discussion

The properties of the cell-material interface are determining factors for the successful functions of cells [16]. Thus, the present in vitro study aimed to investigate the features of the architecture of a collagen sponge that is usually employed as a hemostatic agent. The topographic and biological characterization of this novel collagen sponge showed suitable structural characteristics as biomaterial to favor the cellular interaction and the healing of the tissues. The SEM and histological analyses showed a collagen sponge with a 3-D porous structure, where the average pore size was 244.69 ± 80.01 µm. The ethanol method confirmed the high percentage of porosity that resulted in more than 90%. Large pores, channels, and inter-channel communications were reported to enhance wound healing in vivo and cell growth in vitro onto collagen sponges [17,18]. The biological properties were then investigated on human gingival fibroblasts (HGFs), which are the main cells involved in the wound healing process that is fundamental to regenerating the integrity of the skin. The collagen sponge was also investigated with human oral osteoblasts (HOBs) as a potential material to favor bone regeneration. The material showed an excellent porosity of more than 90% and pore size in the 100 to 300 µm. It was reported that large pores, channels, and inter-channel communications enhance wound healing in vivo and cell growth in vitro onto collagen sponges [17,18]. It was observed with SEM that the HGFs and HOBs adhered to the pore walls and were uniformly distributed, covering the entire surface, and exhibited a typical morphology after 3 days for seeding on the collagen sponge. The shape of the cells appeared in a spindle form, and the attached cells created a dense network on the sponge surface. This is in line with a recent study that showed how a hydrogel scaffold with higher porosity favored the attachment and the vitality of chondrocytes compared to a hydrogel with a smaller porous structure [19]. As a suitable biomedical tool, a porous interconnected structure and high porosity are required, which are important for nutrition supply and cell migration [20]. Even though it was not possible to obtain the images with SEM because of the natural degradation of the collagen sponge at 7 days post-seeding, we decided to investigate the biological activities of cells seeded on sponge at 7 and 14 days. Depending on the degree of cross-linking, the collagen sponge can be degraded highly efficiently by several common enzymes in organisms, such collagenases, tryptase, and lysozyme, into some of the degradation products able to elicit a response in cells such as human fibroblasts, and to promote the restoration of tissue structure and functionality [7,21]. HGFs and HOBs seeded on the collagen sponge showed an increment in the growth at the two timing-points compared to control cells, but the rate of proliferation between the two typologies of cells was different. The growth of fibroblasts was significantly higher than the controls. It reached the peak after 3 days of culture, while the osteoblasts proliferation was relatively low at this time point, and the rate reached the peak after 7 days. The micrographs observed at SEM and the fibroblasts and osteoblasts viability indicated that the 3D porous structure of a collagen sponge is favorable for the adhesion and proliferation of cells. The results also showed that collagen sponges can better promote proliferation in HGFs than in HOBs. For osteoblasts, the mineralization activity was studied. First, the level of the best marker of osteoblastic activity, such as ALP, was investigated. The ALP enzyme showed more activity in osteoblasts seeded on the collagen sponge, but the result was not significantly higher compared to the control. Thus, the expression of encoding genes for ALP was investigated at 3, 7 and 14 days. Given that the secretion of osteocalcin by osteoblasts is linked to ALP activity [22], the level of osteocalcin mRNA (OCN) was also evaluated at 3, 7 and 14 days. It was first observed that the collagen sponge had any significant influences on level of genes encoding ALP and OCN at early time points, such as 3 and 7 days post seeding, despite the fact that at 7 days the ALP and OCN expression resulted higher in respect of the control. Instead, at 14 days of culture, it was found that the collagen sponge significantly increased the ALP mRNA levels, as well as the OCN expression. ALP is a characteristic marker of stage 2 (early-middle period) of osteoblasts differentiation. Our results were in line with various studies that reported a greater ALP expression at 14 days post-culture than 7 days post-culture throughout osteoblast differentiation. The typical trend that is reported in these studies showed that the ALP started to be expressed at 7 days and it reached a peak at 14 days and decreased at 21 days [23,24,25]. Thus, as expected, osteocalcin is secreted when the expression of ALP reaches the highest level to maximize the mineralization of the extracellular matrix (ECM). Furthermore, the ability of cells to produce mineral deposits was investigated via Alizarin red staining. It was found that cells seeded on the collagen sponge significantly enhanced alizarin red staining that highlighted mineralized nodules when compared to the control group. The quantification of calcium deposition confirmed this result with a value significantly higher (+64.45 ± 1.39%) in HOBs on the collagen sponge. High levels of osteoblastic differentiation markers ALP, osteocalcin, and calcium deposits, at a mature stage, were demonstrated in the collagen sponge. The SEM images of the collagen sponge, with cells, showed how the porous three-dimensional structures of the sponge promoted intercellular contact and the accumulation of ECM. This is in line with previous studies that demonstrated how the mean pore size should be around (>100 µm), which is at least three times the cell size [26]. The reason is not only that fibroblasts and osteoblasts have an affinity for collagen, which is a fundamental component of ECM, but the biological activities were also due to the highly porous architecture of the collagen sponge. Together, these preliminary experiments showed that this collagen sponge, already used as hemostatic material, possesses a suitable structural characteristic as a biomaterial to favor the cellular interaction and the healing of the tissues, providing a scaffold for tissue ingrowth and playing an active role in tissue organization during the repair. The 3D porous network allows the cells to permeate and perform regenerative functions. Hence, when it is placed into a wound, it allows cells to deposit along the damaged tissues homogenously. The use of a collagen sponge that is able to remain in the post-operative site for at least the time for wound closure and at the same time to interact with fibroblasts and osteoblasts might be essential to reach the re-epithelization phase to conclude the healing process. Indeed, one of the tremendous surgical complications is represented by dry socket, in which bone can remain exposed for days following surgery due to the bone not having been covered by an initial and persistent blood clot or not having been covered by a layer of the vital, persistent, healing epithelium [11]. Considering that success in skin healing requires suitable niches for neovascularization and proliferation and differentiation of the cells involved in regeneration, the next step might be the investigation of this collagen sponge with highly porous architecture as a scaffold for endothelial cells in creating neovascular structures. In addition, further studies might consider evaluating this collagen sponge as a scaffold for the short-term delivery of antibiotics in the wound bed and to deliver exogenous growth factors. 

## 4. Materials and Methods

### 4.1. Study Design

**Figure 5:**
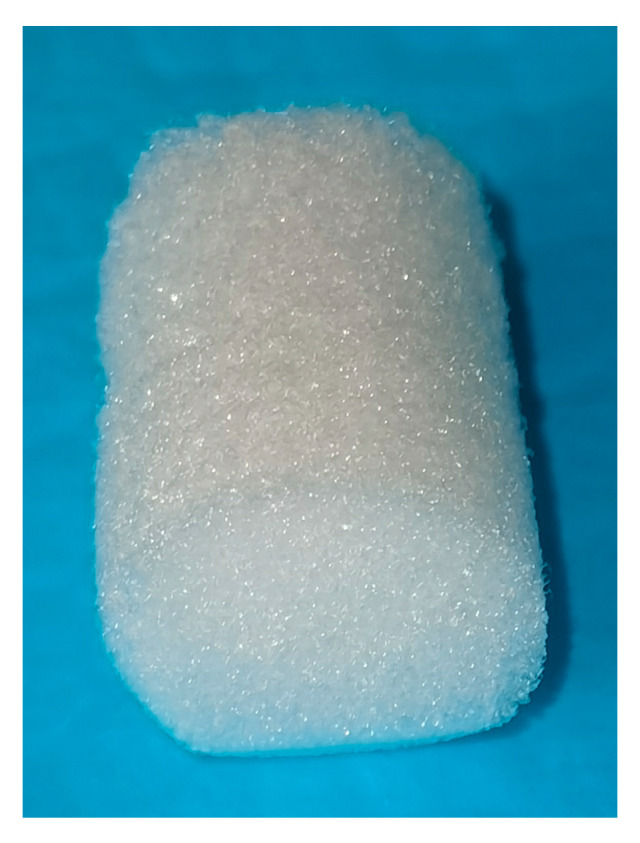
The collagen sponge before to be cut into disks.

The collagen sponge (MVG COLLAGEN PLAST) was produced by MVG Collagen & Technology, Torre Del Lago Puccini, Lucca, Italy). The sponge appeared as a cylindrical block (dimensions: 12 × 8 mm) (Figure 5) that, for the in vitro tests, was cut into disks with a thickness of 3 mm and a diameter of 8 mm.

The collagen sponge disks were firstly characterized in their topography, and then the biological activities were evaluated. Human gingival fibroblasts (HGFs) and oral osteoblasts (HOBs) were cultured on the collagen sponges. The used experimental conditions include cells seeded on the collagen disk (the TEST group) and cells cultured on the bottom of the plate (the CTRL group).

The following analyses were performed:

i. Histological characterization of the sponge alone
ii. SEM characterization of the sponge alone and porosity evaluation
iii. The adhesion of HGFs and HOBs by SEM at 3 and 7 days
iv. Viability of HGFs and HOB by MTT at 3 and 7 days
v. Phosphatase alkaline (ALP) levels of HOBs at 7 days
vi. Mineralization of HOBs at 14 days
vii. Gene expression of constitutive markers of HOBs at 3, 7 and 14 days

### 4.2. Characterization of the Material

#### 4.2.1. SEM Analyses

The SEM observation was performed by using a Desktop-SEM (Phenom-World B.V., Eindhoven, The Netherlands). Before the observation, the samples were fixed with 2.5% glutaraldehyde solution for 1h. They were then dehydrated at increasing alcohol concentrations (40, 50%, 75%, 95%, and 100%) for 10 min each. Samples were then mounted onto aluminum stubs and gold-coated at 150 A with a Desk Sputter Coater (Phenom-World B.V., The Netherlands), as previously described [27]. Images were taken using an accelerating voltage of 15 kV with the backscattered electronic signal detector (BSE), BSD full. The images were taken at 390, 1000, and 3000×.

#### 4.2.2. Determination of Porosity

The porosity of the sponge was studied by the ethanol method and calculated by using the liquid displacement equation [28]:

Porosity (%) = (Wb − Wc − Wd)/(Wa − Wc) × 100%(1)

where Wa a is the quantity of the 10 mL centrifuge tube filled with absolute ethanol, Wb is the total quantity of the 10 mL centrifuge tube with collagen-sponge after its immersion in absolute ethanol, Wc is the remaining mass of absolute ethanol, and the 10 mL centrifuge tube after removal of the sponge, and Wd is the dry weight of the sponge.

#### 4.2.3. Histological Analyses

The specimens were fixed with 10% buffered formalin and dehydrated in an ascending series of alcohol. They were embedded in a glycol methacrylate resin (Technovit 7200 VLC; Kulzer, Wehrheim, Germany) and then polymerized. Sections of about 150 μm were obtained following the long axis of the devices. The sections were subsequently ground down to about 30 μm in width and stained with fuchsin and toluidine blue. The images were taken by optical microscopy (Leica, Wetzlar, Germany) at 40 and 100× magnification. Moreover, the size of the porous was measured.

### 4.3. Biological In Vitro Tests

#### 4.3.1. Cell Cultures

Before cell seeding, sterile sponges were placed in a 24-well culture plates, with one sponge per well. Primary human gingival fibroblasts (HGF) were purchased from ATCC (Manassas, VA, USA) and were cultured in DMEM low glucose (Corning, New York, NY, USA) supplemented with 10% fetal bovine serum (FBS) (SIAL, Rome, Italy), 1% penicillin, and streptomycin (Corning) at 37 °C and 5% CO_2_. Primary human oral osteoblasts (HOB) were extracted) from bone biopsies (Ethical Committee reference numbers: BONEISTO N. 22–10.07.2021) according to the protocol of Pierfelice et al., 2022 [29]. In brief, bone fragments were subjected to three enzymatic digestions at 37 °C for 20, 40 and 60 min using collagenase type 1A (Sigma-Aldrich, St. Louis, MO, USA) and trypsin-EDTA 0.25% (Corning). After each digestion, this solution was centrifuged at 1200 rpm for 10 min, and the pellet obtained was transferred into a T25 culture flask with low-glucose (1 g/L) DMEM supplemented with 10% FBS, 1% antibiotics (100 µg/mL^-1^ streptomycin and 100 IU/mL^-1^ penicillin) and 1% L-glutamine (Corning) at 5% CO_2_ and 37 °C. The cells were used between the third and the fifth passage.

#### 4.3.2. Cell Viability

HGFs and HOBs at the density of 2∙10^4^ cells were seeded on the collagen disks. They were incubated for 3 and 7 days. At the end of each incubation period, the viability was evaluated by MTT assay (Sigma Aldrich, St. Louis, MO, USA,). A solution of 0.5 mg/mL MTT was added to each well, and then the cells were incubated for 4 h at 37 °C and 5% CO_2._ A solution with DMSO was added into each well to dissolve the insoluble formazan. The spectrophotometric absorbance was measured at 650 nm using a microplate reader (Synergy H1 Hybrid BioTek Instruments, Winooski, VT, USA). Five replicates and three independent analyses were assessed.

#### 4.3.3. Cell Adhesion

The samples were dehydrated, gold-sputtered, and observed at SEM, as previously described in Section 2.2.1. The adhesion of cells seeded on the collagen disks were analyzed by SEM at 3 and 7 days. The cells were seeded at a density of 2 × 10^4^ cells/disc for 3 and 7 days at 37 °C in 5% CO_2_. The samples were rinsed twice with 0.1 M PBS (pH 7.4), and the remaining bound cells were fixed as previously explained. The images were taken at 390×.

#### 4.3.4. ALP Assay

5 × 10^4^ HOBs/disk were seeded on the collagen disk in 24-well culture plates for 7 days. The ALP assay kit colorimetric AB83369 (Abcam Inc., Cambridge, UK) was used to evaluate ALP levels. Firstly, the samples were washed three times with PBS and then the cell lysate was obtained. The cell suspension was obtained using a Tissue Rupture device (QIAGEN, Hilden, Germany). This solution was centrifugated at 10,000× *g* for 15 min, and the supernatant was collected. The relative ALP activity was measured following the manufacturer’s instructions. The absorbance was measured at 450 nm by a microplate reader (Synergy H1 Hybrid BioTek Instruments, Winooski, VT, USA).

#### 4.3.5. Alizarin Red Staining and the Quantification of Calcium Deposition

A total of 5 × 10^4^. HOBs/disk were cultured on the collagen disk into the 24-well culture plate, and after 14 days of culture, the cells were fixed with a glutaraldehyde solution (2.5%) for 2 h. The deposits of calcium were evaluated by adding the Alizarin Red staining solution (Sigma-Aldrich, St. Louis, MO, USA) to the culture for 1 h at room temperature. After eliminating the excess dye-deionized water, images were taken by a camera. The intensity of the red color showed the presence of calcium deposits. Next, 1 mL of 10% Cetylpyridinium Chloride (CPC) solution (Sigma-Aldrich, St. Louis, MO, USA) was added to the samples for 1h to chelate the calcium ions. This was used to quantify the calcium nodules. After 1h of incubation, the absorbance was read at 540 nm by a microplate reader (Synergy H1 Hybrid BioTek Instruments, Winooski, VT, USA).

#### 4.3.6. Gene Expression

1 × 10^6^ HOBs were seeded on the collagen disk for 7 and 14 days. Trifast reagents (EuroClone, Pero (MI), Italy) were used to isolate the RNA in accordance with the manufacturer’s instructions. A GoTaq^®^ 2 Step RT-qPCR Kit (Promega, Madison, WI, USA) was used to obtain complementary DNA (cDNA) according to the manufacturer. SYBR Green (GoTaq^®^ 2 Step RT-qPCR Kit, Promega) was used to perform RT-qPCR. 10 µL of mixes, composed by 1 µL of cDNA, 0.2 µL of the primers mixture and 5 µL of master mix were plated in a 96-well plate, and gene expression was determined using the Quant Studio 7 Pro Real-Time PCR System (ThermoFisher, Waltham, MA, USA). The results were normalized to β-actin (β-ACT) for HOB using the 2^−ΔΔCt^ method. Primer sequences are reported in Table 2.

#### 4.3.7. Statistical Analysis

A statistical analysis was performed using GraphPad Prism8 (GraphPad Software, San Diego, CA, USA). A *t*-test was performed to compare the statistical differences between the test group and the control group. The data are presented as the mean ± standard deviation (SD) from at least three biologically repeated experiments, and a *p* < 0.05 was considered to be statistically significant.

## 5. Conclusions

In conclusion, the hemostatic collagen sponge tested in this study exhibited a highly porous structure that favors the adhesion and interconnection of the fibers and promotes the proliferation of gingival fibroblasts and oral osteoblasts. Furthermore, the collagen sponge enhanced the mineralization activities of oral osteoblasts. Further studies are needed to understand the potential of collagen sponges as a scaffold to provide antibiotics and growth factors.

## Figures and Tables

**Table 1:** Pore size in longitudinal sections of the studied scaffolds.

Characteristics	Pore Size (Mean ± SD, µm) (Min–Max)
	Longitudinal (N = 40)
Collagen sponge	244.69 ± 80.01 (106.69–336.36)

**Table 2:** Primer sequences used in RT-qPCR.

Gene	Forward Primer (5′-3′)	Reverse Primer (5′-3′)
OCN ALP	TCAGCCAACTCGTCACAGTC AATGAGTGAGTGACCATCCTGG	GGCGCTACCTGTATCAATGG GCACCCCAAGACCTGCTTTAT
βACT	CCAGAGGCGTACAGGGATAG	GAGAAGATGACCCAGGACTCTC

## Data Availability

MDPI Research Data Policies.
